# Functional Short Tandem Repeat Polymorphism of PTPN11 and Susceptibility to Hepatocellular Carcinoma in Chinese Populations

**DOI:** 10.1371/journal.pone.0106841

**Published:** 2014-09-08

**Authors:** Xiankun Zhao, Shuxiang Hu, Lu Wang, Qing Zhang, Xiaodan Zhu, Hua Zhao, Chaoqun Wang, Ruiyang Tao, Siping Guo, Jing Wang, Jiejie Xu, Yan He, Yuzhen Gao

**Affiliations:** 1 Department of Forensic Medicine, Medical College of Soochow University, Suzhou, Jiangsu, P.R. China; 2 Department of General Surgery, the First Affiliated Hospital of Soochow University, Suzhou, Jiangsu, P.R. China; 3 Key Laboratory of Medical Molecular Virology, MOE & MOH, School of Basic Medical Sciences, Shanghai Medical College, Fudan University, Shanghai, China; 4 Department of Epidemiology, Medical College of Soochow University, Suzhou, Jiangsu, P. R. China; The University of Hong Kong, China

## Abstract

**Background:**

*PTPN11*, which encodes tyrosine phosphatase Shp2, is a critical gene mediating cellular responses to hormones and cytokines. Loss of Shp2 promotes hepatocellular carcinoma (HCC), suggesting that PTPN11 functions as a tumor suppressor in HCC tumorgenesis. The aim of this study was to evaluate the effects of the short tandem repeat (STR) polymorphism (rs199618935) within 3'UTR of *PTPN11* on HCC susceptibility in Chinese populations.

**Methodology/Principal Findings:**

We analyzed the associations in 400 patients from Jiangsu province of China, validating the findings in an additional 305 patients from Shanghai of China. Unconditional logistic regression was used to analyze the association between rs199618935 and HCC risk. Additional biochemical investigations and *in-silico* studies were used to evaluate the possible functional significance of this polymorphism. Logistic regression analysis showed that compared with individuals carrying shorter alleles (11 and 12 repeats), those subjects who carry longer alleles (13 and 14 repeats) had a significantly decreased risk of HCC [adjusted odds ratio (OR)  = 0.63, 95% confidence interval (CI)  = 0.53–0.76, *P* = 2.00×10^−7^], with the risk decreased even further in those carrying allele 15 and 16 (adjusted OR = 0.46, 95% CI = 0.34–0.62, *P* = 1.00×10^−7^). Biochemical investigations showed that longer alleles of rs199618935 conferred higher PTPN11 expression *in vivo* and *in vitro*. The altered luciferase activities in reporter gene system suggested that STR regulation of PTPN11 expression could be a transcriptional event. Finally, *in-silico* prediction revealed that different alleles of rs199618935 could alter the local structure of PTPN11 mRNA.

**Conclusions/Significance:**

Taken together, our findings suggested that the STR polymorphism within *PTPN11* contributes to hepatocarcinogenesis, possibly by affecting PTPN11 expression through a structure-dependent mechanism. The replication of our studies and further functional studies are needed to validate our findings.

## Introduction

Hepatocellular carcinoma (HCC) is one of the most common malignancies and the third leading cause of cancer death [1]. Approximately 80% of HCCs occur in developing countries where hepatitis B virus (HBV) infection is endemic, with the highest incidences being in the Asia-Pacific region, and sub-Saharan Africa [2]. In addition, chronic alcoholism, and long-term exposure to aflatoxin B1 are well-established risk factors for HCC [3]. Molecular biology of carcinogenesis and tumor progression of HCC have been increasingly elucidated with intense research in recent years. However, the molecular and cellular mechanisms of HCC pathogenesis are still poorly understood. Compelling evidence suggests the involvement of host genetic factors in HCC carcinogenesis and genome-wide association studies (GWAS) have greatly contributed to the identification of common genetic variants related to HCC [4], [5]. Thus, it is of particular interest in identifying HCC-related genetic variations, which will definitely benefit the prediction of HCC risks, and the exploration of approaches to prevent HCC development.

Protein tyrosine phosphatase, non-receptor type 11 (*PTPN11*) encodes the non-receptor protein tyrosine phosphatase SHP2, which is critical for RAS/ERK pathway activation in most receptor tyrosine kinase, cytokine receptor, and integrin signaling pathways [6]. SHP2 is widely expressed in most tissues and plays a regulatory role in various cell signaling events that are important for a diversity of cell functions, such as mitogenic activation, metabolic control, transcriptional regulation, and cell migration. Activating mutations in *PTPN11* have been shown to be directly associated with the pathogenesis of Noonan syndrome and childhood leukemias [7], [8]. Several lines of evidence have indicated that PTPN11 is involved in development of multiple cancers [9]–[12], including HCC. PTPN11 is first identified as a proto-oncogene in leukemia [13]. However, most recent findings suggest an unexpected tumor suppressor role of PTPN11 in HCC [14], [15], implying its dual faces in tumorigenesis.

Previous studies have reported genetic variation within *PTPN11*, either dependent or independent of interaction with *helicobacter pylori*, is associated with the risks of gastric cancer and/or atrophic gastritis that precede carcinoma [16]. While the contributions of *PTPN11* polymorphisms to HCC susceptibilities has not been investigated. Considering the important roles of PTPN11 in HCC, we hypothesize that genetic variations in *PTPN11* may modulate its expression thus involve in HCC carcinogenesis. In the current study, we selected one trinucleotide short tandem repeat (STR) polymorphism (rs199618935) and conducted a two-stage case-control study to analyze the genetic effect of the polymorphism on the susceptibilities to HCC in Chinese populations. Additional experimental and *in-silico* studies were used to evaluate the possible functional significance of this polymorphism.

## Materials and Methods

### Ethics Statement

This study was approved by the Ethical Committee of Soochow University. Written informed consent was obtained from each participant before investigation.

### Study Populations

The case-control study was performed on genomic DNA extracted from peripheral blood of 705 newly diagnosed incident HCC cases together with 723 controls after obtaining informed consent. All subjects recruited were unrelated ethnic Han Chinese. For the Jiangsu's case-control study (Panel I), the case series were comprised of 400 HCC patients diagnosed, hospitalized and treated in the affiliated hospitals of Soochow University from 2007 to 2011. For the Shanghai's case-control study (Panel II), 305 HCC patients were recruited at Nantong Tumor Hospital from 2003 to 2005. Controls were cancer-free individuals selected from a community nutritional survey that was conducted in the same regions during the same period as the recruitment of cancer patients. Controls without clinical evidence of liver disease were frequency matched for age (±5 years) and sex to each set of HCC individuals. The diagnosis of the cases, the inclusion and exclusion criteria for the cases and controls, and the definition of smokers and drinkers were described previously in details [17], [18]. Briefly, the diagnosis of these patients was confirmed by a pathological examination combined with positive imaging (Magnetic resonance imaging and/or computerized tomography). Tumor stages were assigned according to a modified American Joint Committee on Cancer (AJCC) and international union against cancer (UICC) standard. The “current smokers” were individuals who had kept smoking almost every day for more than one year till the time of interview; and the “former smokers” were those who experienced the same degree of smoking as the “current smokers”, but stopped smoking at least one year prior to the interview; the non-smokers were those either never smoked or seldom did. Subjects were considered as “light drinkers”, if they consumed 1–2 alcohol drinks per week for more than one year. Those who consumed more than 2 alcohol drinks per week for more than one year were categorized as “heavy drinkers”. “Non-drinkers” were those either never drank or seldom did.

Additional 48 tumor tissues and adjacent non-HCC tissues from patients with a diagnosis of HCC were collected from Department of General Surgery, the First Affiliated Hospital of Soochow University from 2011 to 2012. All cases had histological confirmation of their tumor diagnosis and none of these patients had received preoperative chemotherapy or radiotherapy. After surgical resection, the fresh tissues were immediately stored at −80°C until the DNA/RNA extraction was processed.

### DNA Extraction and Genotyping

Genomic DNA of peripheral blood samples, tissue samples and HCC cell lines were isolated using genomic DNA purification kit (Qiagen). DNA fragments containing rs199618935 were amplified with a pair of genotyping primers (Forward primer: 5′- GTGTCCCTTCTACTTCCCTCT-3′, Reverse primer: 5′- GCTGGGCTTGTGACTTGTTT-3′). The PCR products were analyzed by 7% non-denaturing polyacrylamide gel electrophoresis (PAGE) and visualized by silver staining [19]. For the six different alleles we observed, a direct sequencing method was used to determine the number of repeat motif. The nomenclature of allele was determined according to the recommendations of the DNA commission of the International Society for Forensic Haemogenetics [20]. The genotypes of all samples were analyzed using a homemade allelic ladder, a mixture of all six different alleles. Approximately 10% of the samples were randomly selected and examined in blind duplicates by independent researchers, and the reproducibility was 100%.

### Real-Time RT-PCR Analysis

The Hep-G2, Hep3B and Huh7 hepatoma cell lines were obtained directly from Shanghai Cell Bank of Chinese Academy of Sciences. Cells were cultured in Dulbecco's Modified Eagle's Medium (DMEM) supplemented with 10% fetal bovine serum (FBS) and 1% penicillin-streptomycin at 37°C in a humidified 5% CO2 incubator before RNA extraction. Total RNA was isolated from hepatoma cell lines and tissue samples using RNA isolation kit (Qiagen). cDNA was generated using random primers and Superscript II reverse transcriptase (Invitrogen). A SYBR Green real-time PCR was performed using Roche LightCycler 480 to quantify relative PTPN11 expression in these samples. GAPDH was chosen as the internal control. Primer sequences used for PTPN11 and GAPDH were as follows: PTPN11-F: 5′-TCAGCACAGAAATAGATGTG-3′, PTPN11-R: 5′- TGCTTATCAAAAGGTAGTCA-3′, GAPDH-F: 5′-CTCTCTGCTCCTCCTGTTCGAC-3′, GAPDH-R: 5′-TGAGCGATGTGGCTCGGCT-3′. The 25 µl total volume final reaction mixture consisted of 1 µM of each primer, 12.5 µl of Master Mix (Applied Biosystems), and 50–100 ng of cDNA. The negative control experiments were performed with distilled H_2_O as template. The expression levels of target genes were normalized with GAPDH using a 2−ΔΔCT method [21]. In addition, the melting curve analysis was performed for the PCR products to verify the specificity of primer.

### Construction of Reporter Plasmids and Luciferase Assays

The partial structures (∼570 bp) of human PTPN11-3′UTR containing allele 12, 14 and 15 of rs199618935 were amplified with forward primer 5′-GATCTCTAGACCCCAACTGTTAGTCAATCTGAGC-3′ and reverse primer 5′- CATGGATCCTTGTCCCAGCTACTGTAAGCAGC-3′ from three homozygous human genomic DNA samples. The PCR products were separated in agarose gel and extracted, purified, and cloned with TA cloning Kit (Promega). The repeat numbers of different alleles were confirmed by sequencing. Finally, the 3′UTR of Renilla luciferase in the vector pRL-SV40 (Promega) was replaced with the cloned 3′UTR of *PTPN11* by restriction enzymes XbaI and BamHI. The resulting constructs were verified by sequencing.

The Hep-G2, Hep3B, sk-Hep-1 and Huh7 hepatoma cell lines were seeded at 1×10^5^ cells per well in 24-well plates (BD Biosciences). Twenty-four hours after the plating, cells were transfected by Lipofectamine 2000 according to manufacturer's manual. In each well, 500 ng constructed pRL-SV40 vector and 50 ng pGL3 control vector were cotransfected. Six replicates were performed for each group and each experiment was repeated at least three times. After transfection for 24 hr, cells were harvested by the addition of 100 µl passive lysis buffer. Renilla luciferase activities in cell lysate were measured with the Dual Luciferase assay system (Promega) in TD-20/20 luminometer (Turner Biosystems) and were normalized with the firefly luciferase activities.

### *In-silico* Predicting Effects of STR Polymorphism on PTPN11 Folding Structures

As certain conserved structures more likely serve important biological functions, a 60-bp region covering the polymorphism was analyzed using RNAfold to predict the putative influence of different alleles on local folding structures of PTPN11 using default parameters [22].

### Statistical Analysis

The genotype distribution was analyzed by Hardy-Weinberg equilibrium using χ^2^ test. Since rs199618935 is a multi-allele polymorphism, the genotypic frequencies were calculated by a specific counting method based on different alleles. The samples would be classified into specific genotypic groups provided it has one or two specific alleles. Genotypic and allelic frequencies for each allele between HCC patients and controls were compared by χ^2^ test. To facilitate analysis, alleles with frequencies lower than 3% were combined with the adjacent alleles (e.g. allele 11 and 12, allele 13 and 14, allele 15 and 16). Unconditional logistic regression was used to analyze the association between rs199618935 and HCC risk, adjusted by gender, age, smoking, drinking and HBV infection status. As HBV infection was one of the major risk factors, a stratified analysis by HBV infection status for overall population was performed using binary logistic regression model. Student's *t* test was used to examine the differences in luciferase reporter gene expression. The normalized expression values of PTPN11 in tissue samples and hepatoma cell lines were compared with student's *t* test and one-way ANOVA, respectively. These statistical analyses were implemented in Statistic Analysis System software (version 8.0, SAS Institute). *P*<0.05 was used as the criterion of statistical significance. All statistical tests were two sided.

## Results

### The Associations of STR Polymorphism with HCC Susceptibility

**Figure 1:**
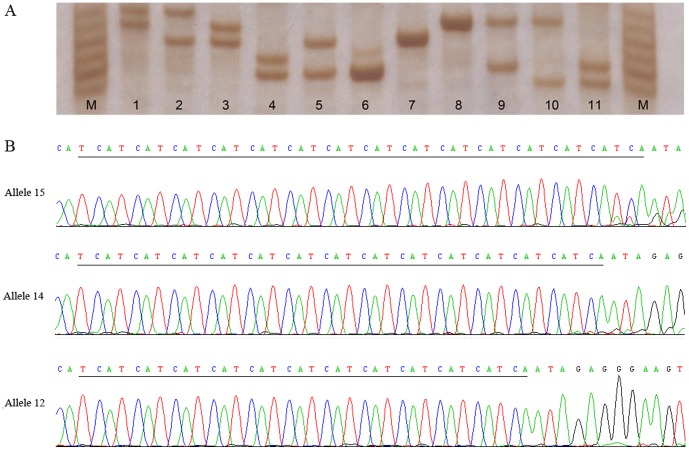
Example sequencing and genotyping output for rs199618935. The upper panel showed an example of the genotyping assay results. DNA was run in 7% non-denaturing polyacrylamide gel electrophoresis (PAGE) and visualized by silver staining (the genotypes from lane 1 to 11 was 15/16, 14/16, 14/15, 12/13, 12/14, 12/12, 14/14, 15/15, 12/15, 11/15 and 11/12, respectively). The lower panel displayed the sequences of the most three common alleles (allele 12, 14 and 15). The underlined base-pairs indicated the “TCA” repeat polymorphism.

The demographic characteristics of the 705 HCC patients and 723 controls from two independent case-control sets were summarized in Table 1. There were no statistically significant differences in terms of the frequency distribution of sex, age, smoking and drinking status, suggesting that the frequency matching was adequate. Approximately 73.0% of the cases and 10.0% of the controls were HBsAg-positive, in accordance with the fact that HBV infection was a major risk factor for HCC. Example output from sequencing and genotyping assay of the STR polymorphism were shown in Figure 1. The observed genotypic frequencies for rs199618935 were consistent with those expected from the Hardy-Weinberg equilibrium in both cases and controls (all *P* values >0.05).

**Table 1:** Demographic characteristics among HCC cases and controls.

Characteristics	Overall			Jiangsu (Panel I)			Shanghai (Panel II)		
	Case	Control	*P*	Case	Control	*P*	Case	Control	*P*
	(n = 705)	(n = 723)		(n = 400)	(n = 408)		(n = 305)	(n = 315)	
Age(mean±SD)	49.8±11.7	51.1±11.6	0.45^a^	52.8±12.1	52.5±11.9	0.48^a^	49.2±10.7	50.5±11.5	0.42^a^
Gender, N (%)									
Male	462(0.66)	488(0.68)	0.43^b^	261(0.65)	274(0.67)	0.57^b^	201(0.66)	214(0.68)	0.5^b^
Female	243(0.34)	235(0.32)		139(0.35)	134(0.33)		104(0.34)	101(0.32)	
Smoking Status									
Nonsmokers	485(0.69)	510(0.71)	0.47^b^	276(0.69)	286(0.70)	0.68^b^	209(0.69)	224(0.71)	0.53^b^
Former Smokers	106(0.15)	104(0.14)		59(0.15)	60(0.15)		47(0.15)	44(0.14)	
Current smoker	114(0.16)	109(0.15)		65(0.16)	62(0.15)		49(0.16)	47(0.15)	
Drinking status									
Nondrinker	392(0.56)	410(0.57)	0.27^b^	217(0.54)	224(0.55)	0.45^b^	175(0.57)	186(0.59)	0.43^b^
Light Drinker	186(0.26)	206(0.28)		113(0.28)	126(0.31)		73(0.24)	80(0.25)	
Heavy Drinker	127(0.18)	107(0.15)		70(0.18)	58(0.14)		57(0.19)	49(0.16)	
Tumor stages									
Ia+Ib	487(0.69)			275(0.69)			212(0.70)		
IIa+IIb	156(0.22)			85(0.21)			71(0.23)		
IIIa+IIIb	62(0.09)			40(0.10)			22(0.07)		
HBsAg, N (%)									
Positive	517(0.73)	71(0.10)	<0.0001^b^	296(0.74)	42(0.10)	<0.0001^b^	221(0.72)	29(0.09)	<0.0001^b^
Negative	188(0.27)	652(0.90)		104(0.26)	366(0.90)		84(0.28)	286(0.91)	

Cases indicate patients with HCC and controls are non-cancerous patients.

a Two-sided two-sample *t*-test between cases and controls.

b χ^2^ test for differences between cases and controls.

Six different alleles (11, 12, 13, 14, 15 and 16) were detected corresponding to 11–16 repeats (allele nomenclature rule is described in methods) and there were totally 11 and 12 different genotypes observed in overall cases and controls, respectively. Genotypic and allelic frequencies of rs199618935 as well as its associations with HCC susceptibility were presented in Table 2. The carriage of allele 11 and 12 was significantly more common (72.5%) in HCC patients, whereas allele 13, 14, 15 and 16 was more common (37.9%) in controls. For the Jiangsu's case-control study, compared with the 11/12 genotype, subjects of 13/14 or 15/16 genotypes of rs199618935 had a significantly decreased risk of HCC in a dose dependent manner (adjusted OR = 0.76, 95%C.I. = 0.58–0.99; adjusted OR = 0.52, 95%C.I. = 0.35–0.79, respectively). Similar trends were observed in the Shanghai's case-control study. Furthermore, based on HBV stratification analysis, no obvious difference was observed between HBV positive and negative population (Table 3).

**Table 2:** Genotypic and allelic frequencies of the trinucleotide STR in HCC patients and controls.

Panel	Repeat number	Genotypic frequency				OR (95% C.I.)^a^	*P*	Allelic frequency				OR (95% C.I.)	*P*
		Cases	%	Controls	%			Cases	%	Controls	%		
Panel I	11/12	375	93.8	341	83.6	1.00 (Reference)		580	72.5	506	62.0	1.00 (Reference)	
	13/14	160	40.0	192	47.1	0.76(0.58–0.99)	0.034	175	21.9	228	27.9	0.67(0.53–0.85)	6.19×10^−4^
	15/16	45	11.3	78	19.1	0.52(0.35–0.79)	1.22×10–^3^	45	5.6	82	10.0	0.48(0.32–0.71)	1.26×10^−4^
Panel II	11/12	287	94.1	260	82.5	1.00 (Reference)		450	73.8	382	60.6	1.00 (Reference)	
	13/14	115	37.7	153	48.6	0.68(0.50–0.92)	0.010	127	20.8	183	29.0	0.59(0.45–0.77)	8.04×10^−5^
	15/16	33	10.8	62	19.7	0.48(0.30–0.78)	1.42×10–^3^	33	5.4	65	10.3	0.43(0.27–0.68)	1.31×10^−4^
Overall	11/12	662	93.9	601	83.1	1.00 (Reference)		1030	73.0	888	61.4	1.00 (Reference)	
	13/14	275	39.0	345	47.8	0.72(0.59–.088)	1.01×10–^3^	302	21.4	411	28.4	0.63(0.53–0.76)	2.00×10^−7^
	15/16	78	11.1	140	19.3	0.51(0.37–0.69)	5.70×10–^6^	78	5.5	147	10.2	0.46(0.34–0.62)	1.00×10^−7^

a Adjusted for sex, age, smoking status, drinking status and HBV infection.

**Table 3:** Logistic regression analyses for the association between rs199618935 and risk of HCC in HBV positive and negative groups.

Genotype	HBV positive					HBV negative				
	Case	%	Control	%	OR^a^ (95% c.i.)	Case	%	Control	%	OR a (95% c.i.)
11/12	480	92.8	59	83.1	1.00(Reference)	173	92.0	538	82.5	1.00(Reference)
13/14	198	38.3	33	46.5	0.79(0.49–1.28)	73	38.8	313	48.0	0.74(0.54–1.02)
15/16	66	12.8	15	21.1	0.60(0.31–1.19)	24	12.8	128	19.6	0.61(0.38–1.00)
*P*_trend_					*P* = 0.086					*P* = 0.011

a Adjusted for sex, age, smoking status, drinking status and HBV infection.

### The Genotype-Phenotype Correlations Between the STR Polymorphism and *PTPN11* Expression

**Figure 2:**
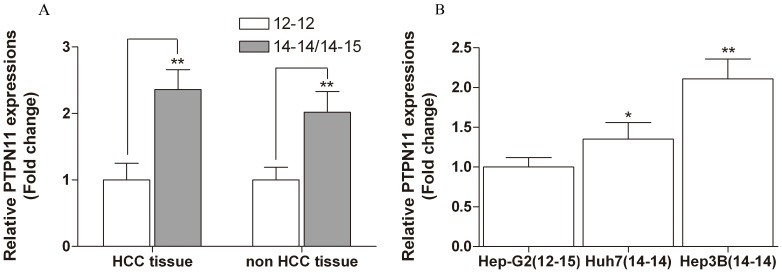
Expression of PTPN11 in HCC tissues and cell lines with different genotypes. (A) PTPN11 mRNA expression (mean ±SEM.) in HCC tissue and adjacent non-HCC tissues samples, by rs199618935 genotype. N = 39 for genotype 12–12, N = 5 for genotype 14–14, N = 4 for genotype 14–15. ** indicates *P*<0.01 compared with 12–12 genotype within the same group. (B) PTPN11 mRNA expression (mean ±SD) in cell lines with different rs199618935 genotypes. * indicates *P*<0.05, ** indicates *P*<0.01 compared with Hep-G2 carrying one 12 allele.

**Figure 3:**
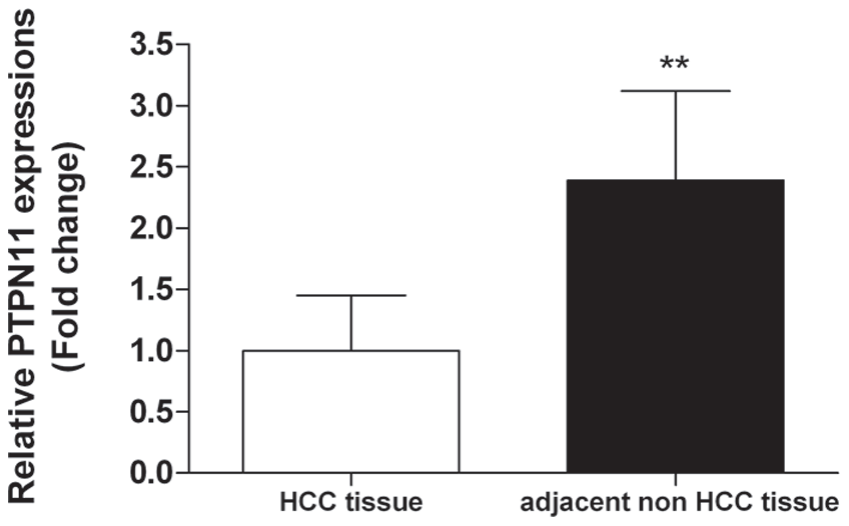
Relative PTPN11 expression in HCC tumor tissues *vs.* adjacent non-tumor tissues. ** indicates *P*<0.01 compared with paired HCC tumor tissue. N = 48.

To further explore the effect of rs199618935 on the expression of PTPN11, we used different genotypic HCC tissue samples as well as their adjacent non-tumor tissues to examine PTPN11 expression. As shown in Figure 2A, results of q-PCR demonstrated that the PTPN11 expression level was significantly correlated with the genotypes of the STR polymorphism. Compared with 12–12 genotype, the PTPN11 expression of 14–14 or 14–15 genotypes was dramatically increased in both HCC tissues and adjacent non-tumor tissues (fold change  = 2.36 and 2.02, respectively, *P*<0.01). To validate our findings in tissue samples, we further examined the genotype-phenotype correlations in three common hepatoma cell lines (Huh7, Hep3B and Hep-G2). Compared with Hep-G2 cell lines carrying 12–15 genotype, the PTPN11 mRNA expression levels of Huh-7 (14–14 genotype) and Hep3B (14–14 genotype) were significantly increased (Figure 2B). Thus, we observed a differential PTPN11 expression pattern in a STR genotype-dependent manner *in vivo* and *in vitro*. Finally, the expression level of PTPN11 in adjacent non-tumor tissues was 2.39-fold higher than that of HCC tumor tissues (Figure 3).

### Effects of the STR Polymorphism on Transcriptional Activity

**Figure 4:**
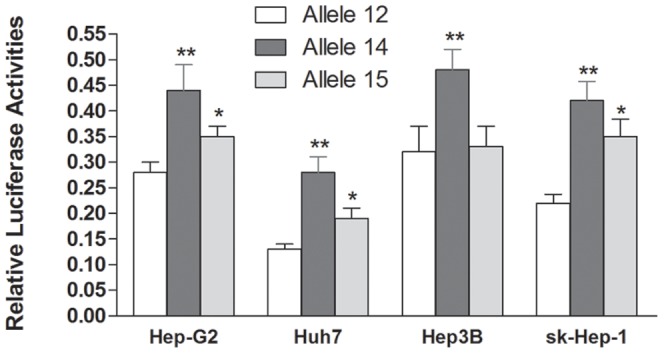
The effects of rs199618935 on PTPN11 transcriptional activity as determined by luciferase reporter assay. In three tested cell lines, significant difference was observed for the relative luciferase activity among allele 12, 14 and 15 constructs. * indicates *P*<0.05, ** indicates *P*<0.01 compared with construct containing allele 12 within the same group.

We further investigated the molecular mechanism underlying correlations between the STR polymorphism and PTPN11 expression. Since rs199618935 was located within 3′UTR of *PTPN11*, two luciferase reporter gene constructs were framed by PCR, and they were used to transiently transfect HCC cell lines. As shown in Figure 4, we found that the constructs containing allele 14 and allele 15 drove an increased reporter expression compared with the constructs containing allele 12 in all four hepatoma cell lines. Of note, allele 14 displayed the highest luciferase expression, which was significantly higher than that of allele 12 and allele 15.

### *In-silico* Analysis of the STR Polymorphism on PTPN11 Folding

**Figure 5:**
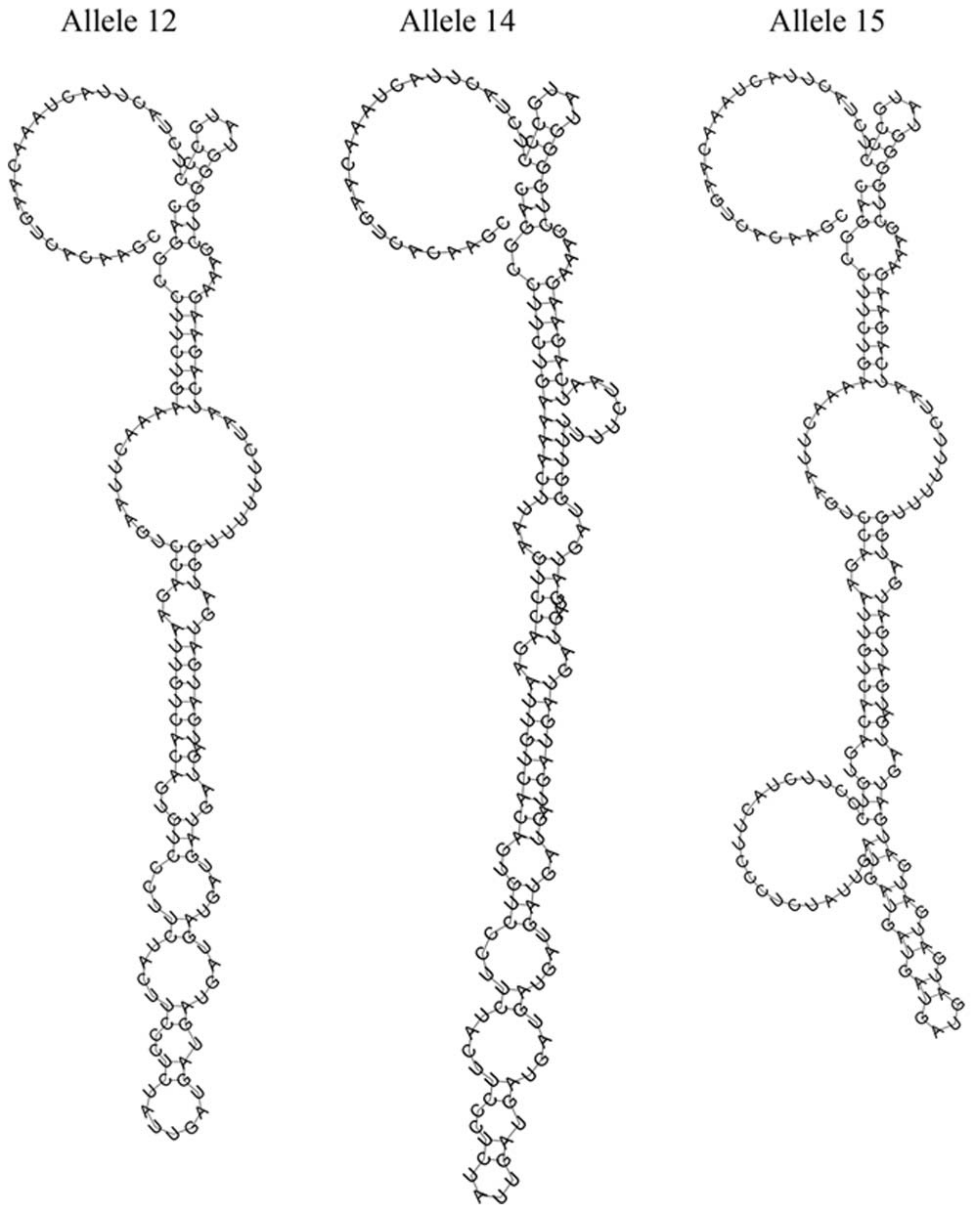
Influence of rs199618935 on PTPN11 mRNA local folding structures. The local structure changes were illustrated by RNAfold using default parameters.

Considering the fact that the STR polymorphism is located within 3′UTR of *PTPN11*, it is plausible that different allele may affect the folding structures of PTPN11, which in turn influence its expression through a structure-dependent mechanism. Using RNAfold algorithms, we predicted the local structure changes of PTPN11 caused by different alleles. As shown in Figure 5, the different “TCA” repeat motif displayed different local structures. Specifically, the allele 12 and 15 appeared to disrupt a highly base paired region which could be formed by the allele 14.

## Discussion

We presented here the first case control study evaluating the association between the novel STR polymorphism within 3′UTR of *PTPN11* and HCC susceptibility. On the basis of our current findings, we propose a schematic model to illustrate the molecular mechanism and functional basis for polymorphism-associated hepatocarcinogenesis conferred by PTPN11 expression. Therefore, the novel STR polymorphism may serve as a potential marker for genetic susceptibility to HCC in Chinese populations.

Most recent experimental data suggest that in contrast to the proto-oncogene effect of dominant-active mutants, PTPN11 may act as a tumor suppressor in hepatocarcinogenesis [14]. Further studies confirmed the tumor suppressor roles of PTPN11 in HCC tumorgenesis and decreased PTPN11 expression has been shown to be a prognostic marker in HCC [15]. Our results also demonstrated that PTPN11 expression was significantly higher in self-matched adjacent non-tumor tissues compared with that of HCC tissues (*P*<0.01), which validated the tumor-inhibiting effect of PTPN11 in HCC. Similarly, Ptpn11 has also been proved to be a tumor suppressor in cartilage and involved in metachondromatosis by inducing hedgehog signaling [23]. Given the critical link between protein-tyrosine kinases (PTKs) activation and oncogenesis, the opposing functions of PTPN11 within different cellular context remain to be fully elucidated.

To test whether the different allele in human *PTPN11* 3′UTR regulates mRNA level, we transfected three different constructs containing different alleles (e.g. 12, 14 and 15) into Huh7, Hep3B, sk-Hep-1 and Hep-G2 cells and then assayed luciferase levels. Our data provided first evidence that the construct containing allele 14 displayed the highest luciferase activity, which was significantly higher than that of constructs containing allele 12 and 15. The altered luciferase activities we observed in the reporter gene system suggested that STR regulation of PTPN11 expression can be a transcriptional event, such as changed RNA stability in a post-transcriptional level. Early studies have shown that STR polymorphism in the 3′UTR formed structural elements (stem-loops) and contributed to mRNA regulation [24]. Indeed, our *in-silico* studies have shown different structures formed by sequences containing different alleles (Figure 5). Based on the results of our current study, we hypothesized that different alleles of the STR may act as an enhancer or repressor to regulate PTPN11 gene expression. Coordinately controlled by PTKs and protein tyrosine phosphatases (PTPs), PTPN11 is a feature of many important signaling pathways that are involved in cell proliferation, adhesion, and migration [25], [26]. Our results showed that longer alleles (14 and 15) conferred higher PTPN11 expression, which was consistent with the fact that longer alleles were associated with decreased HCC risks.

Deregulation of PTPN11 causes hyperactivation of ERK, leading to growth abnormality. Gain-of-function *PTPN11* mutations have been found in various types of human cancer [27]–[29]. It has been shown that the activating SHP2 mutant promotes lung tumorigenesis [30]. Additional data suggest that targeting SHP2 may represent an effective strategy for treatment of epidermal growth factor receptor (EGFR) inhibitor resistant non-small cell lung cancer [31]. Therefore, the STR polymorphism identified in our study may serve as a potential marker for individualized diagnosis or treatment of cancers.

Although this is the first report for the possible association between *PTPN11* STR polymorphism and HCC risk, the significance of this finding is limited by the relative small sample size used in this study. However, result from our genetic association analysis provides a preliminary data and evokes the need for future study with different or expanded case-control populations to confirm our observations. Furthermore, the underlined molecular mechanisms between the STR polymorphism and PTPN11 expression still need to be fully investigated both at genetic and functional levels.

In summary, we have provided initial evidence that the length variation of the “TCA” repeats within human PTPN11 3′UTR may play a functional role in regulating the expression of PTPN11 and subsequently affect development of HCC. Therefore, PTPN11 may be a promising marker for personalized diagnosis and therapy of HCC.

## References

[1] Yang JD, Roberts LR (2010) Hepatocellular carcinoma: A global view. Nat Rev Gastroenterol Hepatol 7: 448–458. 20628345 10.1038/nrgastro.2010.100 PMC3926946

[2] El-Serag HB (2012) Epidemiology of viral hepatitis and hepatocellular carcinoma. Gastroenterology 142: 1264–1273. 22537432 10.1053/j.gastro.2011.12.061 PMC3338949

[3] Monto A, Wright TL (2001) The epidemiology and prevention of hepatocellular carcinoma. Semin Oncol 28: 441–449. 11685737 10.1016/s0093-7754(01)90137-x

[4] Kumar V, Kato N, Urabe Y, Takahashi A, Muroyama R, et al (2011) Genome-wide association study identifies a susceptibility locus for HCV-induced hepatocellular carcinoma. Nat Genet 43: 455–458. 21499248 10.1038/ng.809

[5] Clifford RJ, Zhang J, Meerzaman DM, Lyu MS, Hu Y, et al (2010) Genetic variations at loci involved in the immune response are risk factors for hepatocellular carcinoma. Hepatology 52: 2034–2043. 21105107 10.1002/hep.23943 PMC8259333

[6] Grossmann KS, Rosário M, Birchmeier C, Birchmeier W (2010) The tyrosine phosphatase Shp2 in development and cancer. Adv Cancer Res 106: 53–89. 20399956 10.1016/S0065-230X(10)06002-1

[7] Tartaglia M, Gelb BD (2005) Germ-line and somatic PTPN11 mutations in human disease. Eur J Med Genet 48: 81–96. 16053901 10.1016/j.ejmg.2005.03.001

[8] Loh ML, Vattikuti S, Schubbert S, Reynolds MG, Carlson E, et al (2004) Mutations in PTPN11 implicate the SHP-2 phosphatase in leukemogenesis. Blood 103: 2325–2331. 14644997 10.1182/blood-2003-09-3287

[9] Mohi MG, Neel BG (2007) The role of Shp2 (PTPN11) in cancer. Curr Opin Genet Dev 17: 23–30. 17227708 10.1016/j.gde.2006.12.011

[10] Leibowitz MS, Srivastava RM, Andrade Filho PA, Egloff AM, Wang L, et al (2013) SHP2 is overexpressed and inhibits pSTAT1-mediated APM component expression, T-cell attracting chemokine secretion, and CTL recognition in head and neck cancer cells. Clin Cancer Res 19: 798–808. 23363816 10.1158/1078-0432.CCR-12-1517 PMC3578140

[11] Liu X, Zheng H, Qu CK (2012) Protein tyrosine phosphatase Shp2 (Ptpn11) plays an important role in maintenance of chromosome stability. Cancer Res 72: 5296–5306. 22890240 10.1158/0008-5472.CAN-12-1495 PMC3473170

[12] Chang W, Gao X, Han Y, Du Y, Liu Q, et al. (2013) Gene expression profiling-derived immunohistochemistry signature with high prognostic value in colorectal carcinoma. Gut (doi: 10.1136/gutjnl-2013-305475). 10.1136/gutjnl-2013-305475 24173294

[13] Chan RJ, Feng GS (2007) PTPN11 is the first identified proto-oncogene that encodes a tyrosine phosphatase. Blood 109: 862–867. 17053061 10.1182/blood-2006-07-028829 PMC1785139

[14] Bard-Chapeau EA, Li S, Ding J, Zhang SS, Zhu HH, et al (2011) Ptpn11/Shp2 acts as a tumor suppressor in hepatocellular carcinogenesis. Cancer Cell 19: 629–639. 21575863 10.1016/j.ccr.2011.03.023 PMC3098128

[15] Jiang C, Hu F, Tai Y, Du J, Mao B, et al (2012) The tumor suppressor role of Src homology phosphotyrosine phosphatase 2 in hepatocellular carcinoma. J Cancer Res Clin Oncol 138: 637–646. 22228034 10.1007/s00432-011-1143-5 PMC11824761

[16] He C, Tu H, Sun L, Xu Q, Li P, et al (2013) Helicobacter pylori-related host gene polymorphisms associated with susceptibility of gastric carcinogenesis: a two-stage case-control study in Chinese. Carcinogenesis 34: 1450–1457. 23455381 10.1093/carcin/bgt079

[17] Zhu Z, Gao X, He Y, Zhao H, Yu Q, et al (2012) An insertion/deletion polymorphism within RERT-lncRNA modulates hepatocellular carcinoma risk. Cancer Res 72: 6163–6172. 23026137 10.1158/0008-5472.CAN-12-0010

[18] Wan J, Huang M, Zhao H, Wang C, Zhao X, et al (2013) A novel tetranucleotide repeat polymorphism within KCNQ1OT1 confers risk for hepatocellular carcinoma. DNA Cell Biol 32: 628–634. 23984860 10.1089/dna.2013.2118

[19] Allen RC, Graves G, Budowle B (1989) Polymerase chain reaction amplification products separated on rehydratable polyacrylamide gels and stained with silver. Biotechniques 7: 736–744. 2483661

[20] Lincoln PJ (1997) DNA recommendations–further report of the DNA Commission of the ISFH regarding the use of short tandem repeat systems. Forensic Sci Int 87: 181–184. 9248037

[21] Livak KJ, Schmittgen TD (2001) Analysis of relative gene expression data using real-time quantitative PCR and the 2(-Delta Delta C(T)) Method. Methods 25: 402–408. 11846609 10.1006/meth.2001.1262

[22] Gruber AR, Lorenz R, Bernhart SH, Neuböck R, Hofacker IL (2008) The Vienna RNA Nucleic Acids Res 36(Web Server issue): W70–74. 10.1093/nar/gkn188 PMC2447809 18424795

[23] Yang W, Wang J, Moore DC, Liang H, Dooner M, et al (2013) Ptpn11 deletion in a novel progenitor causes metachondromatosis by inducing hedgehog signalling. Nature 499: 491–495. 23863940 10.1038/nature12396 PMC4148013

[24] Mooers BH, Logue JS, Berglund JA (2005) The structural basis of myotonic dystrophy from the crystal structure of CUG repeats. Proc Natl Acad Sci USA 102: 16626–16631. 16269545 10.1073/pnas.0505873102 PMC1283809

[25] Chan G, Kalaitzidis D, Neel BG (2008) The tyrosine phosphatase Shp2 (PTPN11) in cancer. Cancer Metastasis Rev 27: 179–192. 18286234 10.1007/s10555-008-9126-y

[26] Feng GS (2012) Conflicting roles of molecules in hepatocarcinogenesis: paradigm or paradox. Cancer Cell 21: 150–154. 22340589 10.1016/j.ccr.2012.01.001 PMC3285429

[27] Bentires-Alj M, Paez JG, David FS, Keilhack H, Halmos B, et al (2004) Activating mutations of the noonan syndrome-associated SHP2/PTPN11 gene in human solid tumors and adult acute myelogenous leukemia. Cancer Res 64: 8816–8820. 15604238 10.1158/0008-5472.CAN-04-1923

[28] Taylor BS, Schultz N, Hieronymus H, Gopalan A, Xiao Y, et al (2010) Integrative genomic profiling of human prostate cancer. Cancer Cell 18: 11–22. 20579941 10.1016/j.ccr.2010.05.026 PMC3198787

[29] Ding L, Getz G, Wheeler DA, Mardis ER, McLellan MD, et al (2008) Somatic mutations affect key pathways in lung adenocarcinoma. Nature 455: 1069–1075. 18948947 10.1038/nature07423 PMC2694412

[30] Schneeberger VE, Luetteke N, Ren Y, Berns H, Chen L, et al. (2014). SHP2E76K mutant promotes lung tumorigenesis in transgenic mice. Carcinogenesis 2014 (in press). 10.1093/carcin/bgu025 PMC4123642 24480804

[31] Xu J, Zeng LF, Shen W, Turchi JJ, Zhang ZY (2013) Targeting SHP2 for EGFR inhibitor resistant non-small cell lung carcinoma. Biochem Biophys Res Commun 439: 586–590. 24041688 10.1016/j.bbrc.2013.09.028 PMC3822432

